# Female Urinary Incontinence in Africa: Prevalence Estimates from a Systematic Review and Meta-Analysis

**DOI:** 10.1007/s00192-025-06146-6

**Published:** 2025-05-30

**Authors:** Jeanne Bertuit, Andy-Muller Luzolo Nzinga, Véronique Feipel

**Affiliations:** 1 HESAV School of Health Sciences - Vaud, HES-SO University of Applied Sciences and Arts Western, Lausanne, Switzerland; 2 Pelvic Floor Rehabilitation Unit, Department of Physical Medicine and Rehabilitation, University Clinics of Kinshasa, Faculty of Medicine, University of Kinshasa, Kinshasa, Democratic Republic of Congo; 3 Laboratory of Functional Anatomy, Faculty of Motor Sciences, Université Libre de Bruxelles, Brussels, Belgium; 4 Laboratory of Anatomy, Biomechanics and Organogenesis, Faculty of Medicine, Université Libre de Bruxelles, Brussels, Belgium

**Keywords:** Africa, Female, Prevalence, Urinary incontinence

## Abstract

**Introduction:**

This systematic review and meta-analysis aimed to investigate the prevalence of urinary incontinence (UI) among women in African countries. Different types of UI, racial distributions, geographic locations, and methodological approaches were analyzed and compared.

**Methods:**

A systematic search was conducted using CINAHL, PubMed, Embase, and African Journals Online (AJOL). Studies published between 2000 and 2023 in French or English were included if they assessed the prevalence of UI among adult women (≥18 years) in Africa. A meta-analysis using a random-effects model was performed. The PRISMA checklist guided the reporting of this review.

**Results:**

A total of 22 studies were included. The pooled prevalence of UI was 24% (95% CI: 17–33%), with individual study estimates ranging from 2% to 80%. The pooled prevalence was 28% (95% CI: 19–38%) for urgency urinary incontinence (UUI), 35% (95% CI: 26–45%) for stress urinary incontinence (SUI), and 31% (95% CI: 18–45%) for mixed urinary incontinence (MUI). High heterogeneity was observed across studies (I² ranging from 72.6% to 99.8%; p 0.001 for Cochran’s Q test in all UI subcategories).

**Conclusion:**

Urinary incontinence affects approximately one-quarter of adult women in Africa. However, the high heterogeneity in prevalence estimates—related to differences in methodology and UI definitions—limits the ability to draw firm conclusions.

## Introduction

The International Continence Society (ICS) and the International Urogynecological Association (IUGA) have defined urinary incontinence (UI) as “a complaint of involuntary urine leakage” [1]. UI affects more than 200 million individuals worldwide [2]. A recent meta-analysis by Mostafaei et al. reported a prevalence ranging from 2.8% to 57.7% [3]. The total prevalence of UI was 25.7% (95% CI 22.3 to 29.5%). UI leads to a decrease in quality of life, as women may develop a sense of shame and a negative perception of themselves, leading them to restrict or reduce their physical and social activities. UI has a devastating effect on physical, sexual, social, and psychological levels, which can lead to symptoms of depression [4–8]. In addition to these impacts, UI represents a significant financial burden at both the individual and collective health care levels.

Urinary incontinence is classified into three types most common in women: stress urinary incontinence (SUI), which corresponds to involuntary leakage of urine during effort or physical exertion, or coughing and sneezing; urgency urinary incontinence (UUI), which corresponds to involuntary leakage of urine accompanied or immediately preceded by a feeling of urgency; and mixed urinary incontinence (MUI) [1], which corresponds to involuntary leakage of urine associated with a feeling of urgency but also during stress [9–12]. SUI is predominant in women and is considered a common condition. The prevalence rates for stress, urgency, and mixed UI were 12.6% (95% CI 10.3 to 15.4%), 5.3% (95% CI 3.4 to 8.3%), and 9.1% (95% CI 7.0 to 11.8%) respectively [3].

Prevalence is influenced by several factors. When the older adult population was excluded, the UI prevalence changed slightly to 26.2% (95% CI 22.6 to 30.2%). The questions on UI understanding also affected the prevalence, which changed from 25.5% (95% CI 18.5 to 34.2%) to 41.2% (95% CI 18.4 to 68.5%) owing to several definitions of UI. Furthermore, the use of validated or unvalidated questionnaires also impacted on the prevalence, with higher rates reported for unvalidated questionnaires at 27.7% (CI95%: 22.6‐33.4%). Finally, geographical area is another factor that affects prevalence, with higher percentages in the African region, such as the Middle East and North Africa at 37.3% (95% CI 25.8 to 50.5%), and lower prevalence in South Asia at 14.2% (95% CI 6.1 to 29.8%), and the sub-Saharan region at 4.6% (95% CI 1.7 to 12.3%). These variations in prevalence across regions highlight the social impact and different methods of treatment, as well as differences in data collection and processing methodologies [3]. So, it should be noted that prevalence estimates vary according to age, use of validated questionnaires, time frame, or setting.

Investigating disease prevalence across African countries is crucial for advancing scientific knowledge and optimizing health policies. This research will enable precise resource allocation, inform targeted medical interventions, and strengthen health care systems. Consequently, it enhances public health strategies and outcomes, fostering better population health and evidence-based decision making across the continent.

This review was aimed at addressing this critical knowledge gap by synthesizing existing evidence on the prevalence of UI in African countries. By systematically reviewing and analyzing the available data, in this study we seek to provide a robust and comprehensive understanding of the magnitude of UI in diverse African populations. This includes exploring the potential variations in prevalence rates across different geographical regions, age groups, and socioeconomic strata within the continent.

## Materials and Methods

### Protocol

The official PRISMA recommendations were used to carry out and write this literature review. It is registered under ID CRD42023490996 in PROSPERO.

### Eligibility Criteria

In order to refine the search, inclusion and exclusion criteria were determined (Table 1). Studies carried out in Africa on adult females aged 18 years or over with the objective of determining the prevalence or incidence of urinary incontinence (including SUI, UUI, and MUI) were selected. Only quantitative descriptive and epidemiological studies published between 2000 and 2023 in English or French were included. Studies on prolapse, fistulas, neurological bladders, and women who were pregnant or postpartum for 6 months or less were excluded.

**Table 1:** Eligibility criteria

	Inclusion criteria	Exclusion criteria
Population	African adult women	Age < 18 years old
	Age ≥ 18 years old	Pregnant women
		Women with fistula
		Postpartum ≤ 6 months
		Women with known neurological pathological conditions (diabetes, spinal cord injury, etc.)
Outcomes	Prevalence/incidence of urinary incontinence	Fistula
	Prevalence/incidence of stress urinary incontinence	Neurogenic bladder
	Prevalence/incidence of urgency urinary incontinence	
	Prevalence/incidence of overactive bladder	
Language	English and French	
Published date	From 2000 to 2023	
Design of study	Descriptive study Epidemiological study	
	Cross-sectional study	
Study method	Quantitative	
Place of study	Continent of Africa	

### Search Strategy

This study was conducted from August 2023 to March 2024. Three databases were used in this study: CINAHL, PubMed, and Embase. The African Journal Online (JOL), which is the world’s largest online collection of African-published, peer-reviewed scholarly journals, was also used. Keywords and descriptors were chosen to respond best to the topic. The descriptors were determined using the thesaurus of various databases. Subsequently, the use of Boolean operators “OR and AND” allowed us to assemble the keywords and descriptors to create a search equation that was then developed and specified according to each database (Table 2).

**Table 2:** Search equation

Generic search equation	
	Epidemiolog* OR prevalence OR incidence OR magnitude OR burn* OR rate
AND	"Pelvic floor"OR"pelvic floor disorder"OR"pelvic floor dysfunction"OR"urinary incontinence"OR"Urge incontinence"OR"stress incontinence"OR"urine leakage"OR"lower urinary tract disorders"OR"lower urinary tract symptoms"OR"overactive bladder"OR"anorectal symptoms"OR"fecal incontinence"OR"anal incontinence"OR"bowel symptoms"OR"double incontinence"OR"sexual dysfunction"OR dyspareunia OR"chronic pelvic pain"
AND	Wom?n OR Nulliparous OR multiparous OR female
AND	Afric* OR Sub-saharan* OR Algeria* OR Angola* OR Benin* OR Botswan* OR Burkina* OR Burundi* OR"Cape Verde*"OR Cameroon* OR"Central African"OR Chad* OR Congo* OR Kinshasa OR kivu OR"Cote ivoir*"OR"Ivory Coast"OR Djibouti* OR Egypt* OR Eritrea* OR Ethiopia* OR Gabon* OR Gambia* OR Ghana* OR Guinea* OR Kenya* OR Liberia* OR Libya* OR Madagascar* OR Malawi* OR Mali* OR Mauritania* OR Mauriti* OR Morocc* OR Mozambi* OR Namibia* OR Niger* OR Nigeria* OR"Papua New Guinea*"OR Rwanda* OR"Sao Tome*"OR Senegal* OR Seychell* OR"Sierra Leon*"OR Somalia* OR"South Africa*"OR Tanzania* OR Togo* OR Tunisia* OR Uganda* OR Zambia* OR Zimbabwe*

### Study Selection and Data Extraction

Two authors (ANM and JB) independently reviewed the titles and abstracts of studies obtained from the databases. Compliance with the eligibility criteria was assessed. Subsequently, the same authors independently verified, by using a full reading of the studies, the criteria defined in order to include or exclude them from the review. In the case of disagreement, another author helped to decide between situations.

### Methodological Quality Assessment

The *Checklist for Prevalence Studies* by the Joanna Briggs Institute was used for quality assessment. The purpose was to assess the methodological quality of a study and to determine the extent to which a study has addressed the possibility of bias in its design, conduct, and analysis [13]. This checklist has nine items:

1. Was the sample frame appropriate to address the target population?
2. Were study participants sampled in an appropriate way?
3. Was the sample size adequate?
4. Were the study subjects and the setting described in detail?
5. Was the data analysis conducted with sufficient coverage of the identified sample?
6. Were valid methods used for the identification of the condition?
7. Was the condition measured in a standard, reliable way for all participants?
8. Was there appropriate statistical analysis?
9. Was the response rate adequate, and if not, was the low response rate managed appropriately?

All the papers selected in the systematic review were evaluated. The questions with answer “yes” are shown as  , with answers “no” as  , and answer “unclear” as  . A total score greater than 80% was defined as high quality, a score between 60 and 80% as medium quality, and a score less than 60% as low quality.

### Data Analysis

Extracted data included details of specific variables, such as the country where the study was carried out, study design, type of sampling method, calculation and sample size, eligibility criteria, age, study site and geographic categories, method of data collection, validation of assessment tool, definition of UI according to ICS, and prevalence of UI and their types.

### Meta-Analysis

Quantitative analysis was performed on the prevalence of the selected studies. Quantitative paper data were pooled in the statistical meta-analysis using Stata SE software (version 18). A meta-analysis of prevalence was performed using a random-effects model.

The effect size and weighted mean differences for proportions with 95% confidence interval (95% CI) were calculated for the analyses. The *z* test for the overall effect was also calculated. If the *p* value was < 0.05, it was the difference in prevalence between studies.

Heterogeneity was assessed using Cochran’s Q test. When Cochran’s Q test was significant (*p* < 0.05), statistical heterogeneity was present. The I^2^ test was used to assess the inconsistency between studies, with values of 25%, 50%, and 75% corresponding to low, moderate, and high heterogeneity respectively.

If there is a high level of heterogeneity, a random-effects model (DerSimonian and Laird random-effects model = DL) is more appropriate, and the DerSimonian and Laird’s estimator for tau squared (τ^2^ estimate DL) is the method most widely used to estimate the between-study variance. Hence, if τ^2^ estimate is zero, the random effects and fixed effects models are the same.

## Results

### Selection of Studies

**Fig. 1:**
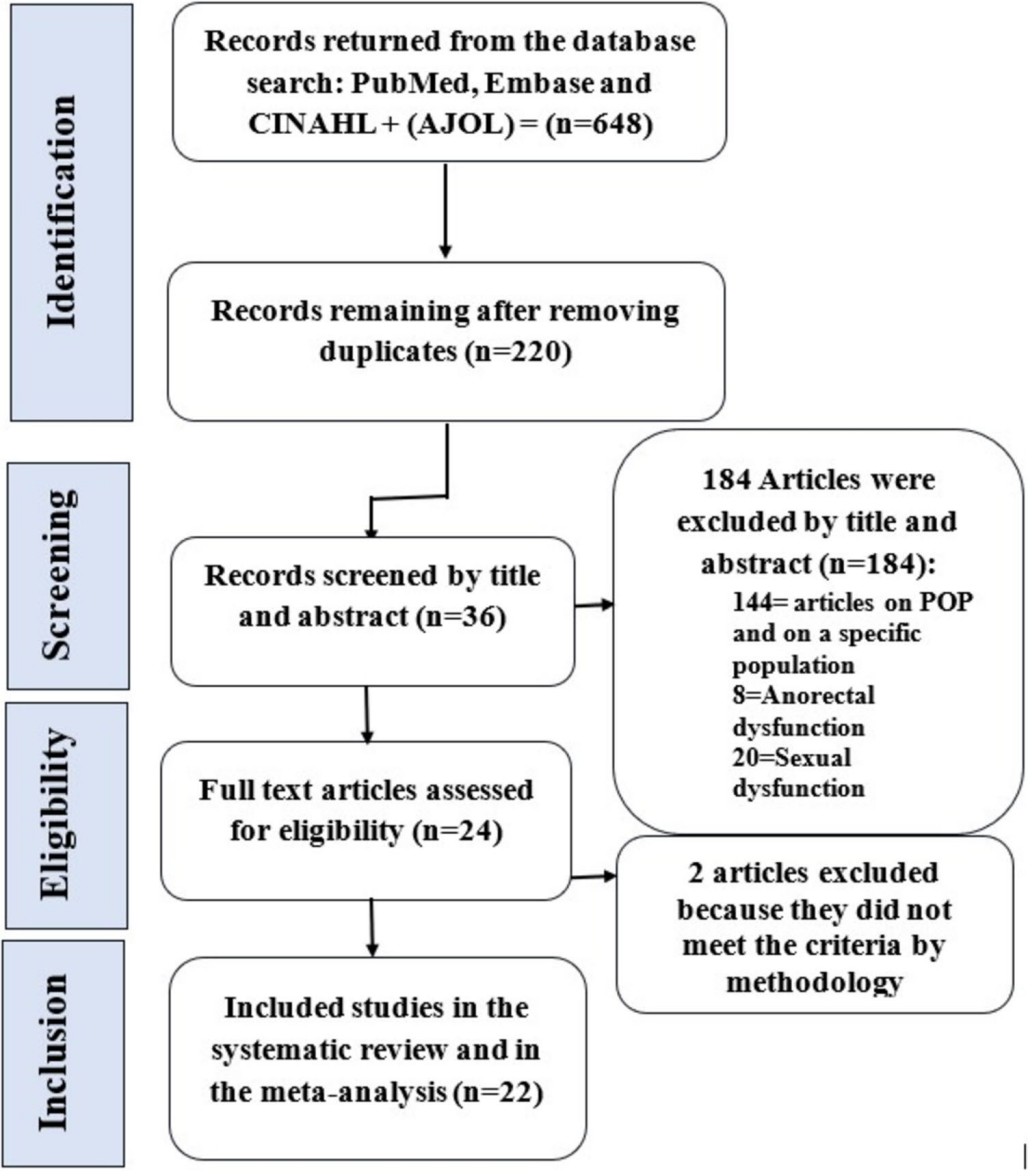
Flow-chart

Database searches identified 648 articles. After removing duplicates (*n* = 428) and selecting articles based on title, abstract, and full text, 22 articles were retained. A summary of the search results and study selection is shown in the PRISMA (Preferred Reporting Items for Systematic Reviews and Meta-Analyses) diagram (Fig. 1).

### Quality

**Fig. 2:**
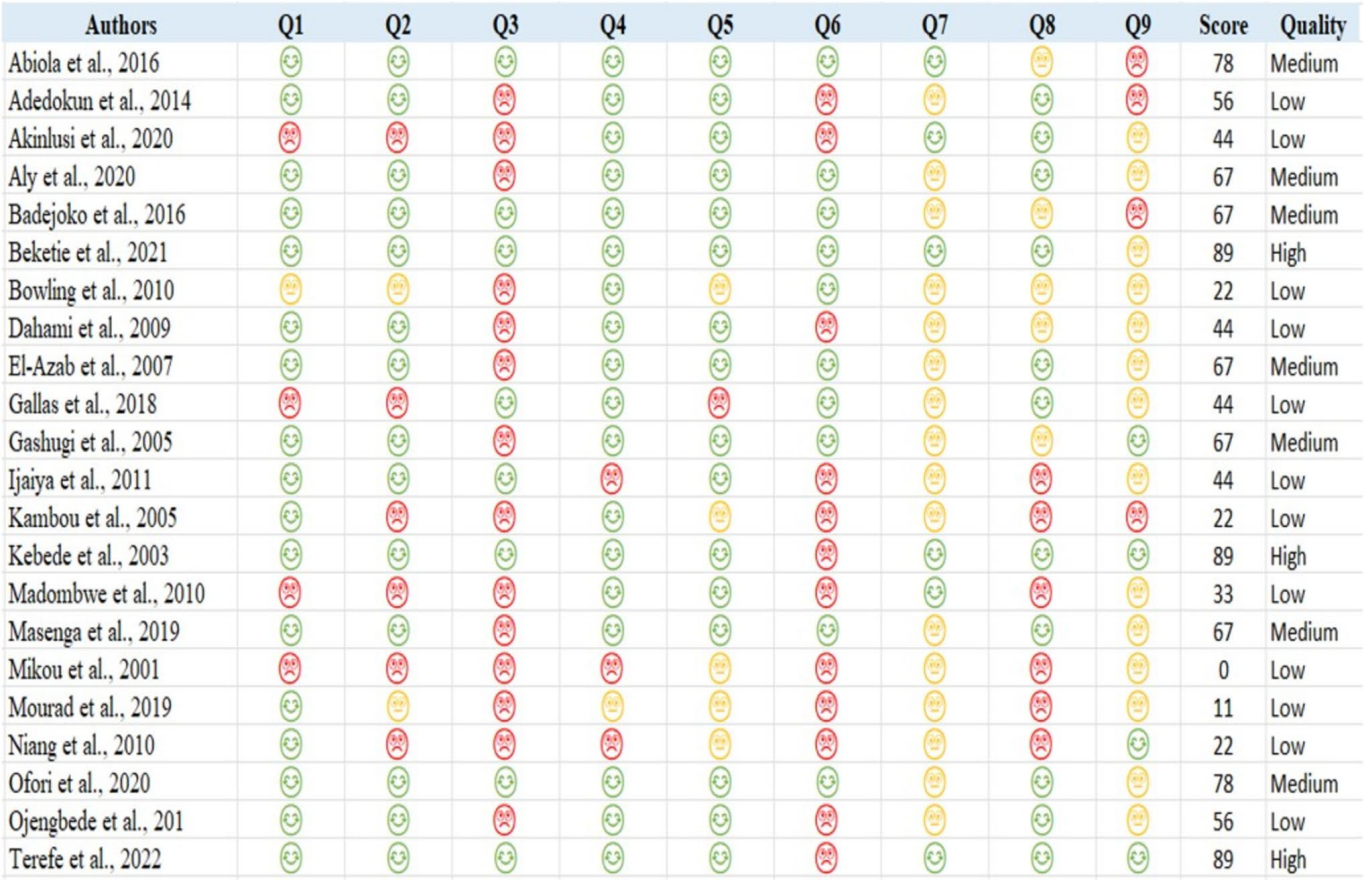
Quality assessment results with the Joanna Briggs Institute checklist for prevalence studies consisting of nine questions

All articles were evaluated using the Checklist for Prevalence Studies (Fig. 2). Of the 22 articles selected, 3 (13.6%) were of high quality, 7 (31.8%) were of medium quality, and 12 (54.6%) were of low quality.

### Characteristics of Studies

The characteristics of the 22 selected articles are illustrated in Table 3.

**Table 3:** Characteristics of studies

Reference	Country	Study design	Type of sampling method	Calculating the sample size	Sample size (*n*)	Criteria	Age (mean or median) (years)	Age group (year)	Study site (geographic categories)	Method of data collection	Validation of assessment tool	UI definition according to ICS	Prevalence (95% CI)			
													UI	UUI	SUI	MUI
Abiola et al. [14]	Nigeria	Cross-sectional study	Random sampling	Yes	229	Women > 18 years old	50 ± 7.23	24 to 74	Community (rural)	Questionnaire	ICIQ-UI SF	No	12.7	27.6	58.6	13.8
Adedokun et al. [19]	Nigeria	Cross-sectional study	Random sampling	No	254	Women > 45 years old	60.3 ± 10.2	45 to 70+	Community (urban)	Questionnaire	No	No	10 (8.1–11.99)	40	28	–
Akinlusi et al. [15]	Nigeria	Cross-sectional study	Nonrandom sampling	No	395	Women who visited the clinic > 25 years old	38.81 ± 10.1	25 to 67	Hospital	Questionnaire	No	No	32.9	55	22	23
Aly et al. [20]	Egypt	Cross-sectional study	Nonrandom sampling	No	130	Frail elderly females > 60 years old	70.7 ± 8.3	≥ 60	Hospital	Questionnaire, clinical examination	ICIQ-UI SF	No	80	0.96	4.8	91
Badejoko et al. [16]	Nigeria	Cross-sectional study	Nonrandom sampling	Yes	1250	Women who visited the clinic > 20 years old	46.8 ± 17.7	20 to 100	Hospital (semi-urban)	Questionnaire	QUID	–	5.2	46.2	33.8	20
Beketie et al. [23]	Ethiopia	Cross-sectional study	Random sampling	Yes	542	Women > 18 years old	Median = 36; (IQR = 20)	19 to 70	Community	Questionnaire	No	IUGA and ICS	32.8	–	–	–
Bowling et al. [30]	Liberia	Cross-sectional study	Nonrandom sampling	No	424	All women	36.5 ± 15.5		Community	Questionnaire	PFDI-20	No	1.7	–	–	–
Dahami et al. [26]	Morocco	Cross-sectional study	Nonrandom sampling	No	338	Women: 20 to 40 years old	–	20 to 40	Community (urban)	Questionnaire	No	–	14.2	27.1	54.2	14.5
El-Azab et al. [21]	Egypt	Cross-sectional study	Random sampling	No	1652	Women > 20 years old	–	≥ 20	Community (rural and urban)	Questionnaire	UDI-6	ICS	54.8	27.4	27.0	45.64
Gallas et al. [28]	Tunisia	Cross-sectional study	Random sampling	Yes	402	Health care professionals > 20 years old	36.8 ± 8.32	23 to 60	Hospital	Questionnaire	USP	–	45	40.3	24.6	19.9
Gashugi et al. [29]	Rwanda	Cross-sectional survey with a short retrospective period of 4 weeks	Random sampling	No	1030	Women > 20 years old	–	20 to 64	Community (urban)	Questionnaire	ICIQ-UI SF	–	42	–	–	–
Ijaiya et al. [17]	Nigeria	Cross-sectional study	Nonrandom sampling	Yes	333	Women who visited the clinic	37.4 ± 4.1	≥ 19	Hospital	Questionnaire	No	No	30.6	35.3	39.2	–
Kambou et al. [31]	Burkina Faso	Epidemiological study: prospective and cross-sectional	Nonrandom sampling	No	759	Women > 18 years old	29.8	≥ 18	Community (urban)	Questionnaire	No	–	21.3	27.6	59.5	12.8
Kebede et al. [25]	Ethiopia	Cross-sectional study	Random sampling	Yes	427	All women	Median = 29; IQR = 24–45	≥ 18	Community (rural and urban)	Questionnaire	No	–	28.6 (24.3–32.8)	–	–	–
Madombwe et al. [32]	Republic of South Africa	Cross-sectional study	Random sampling	No	99	Women > 21 years old	43.7	21 to 76	Community (urban)	Questionnaire	No	ICS	35.4 (25.9–44.8)	2.9	65.7	31.4
Masenga et al. [33]	Tanzania	Cross-sectional study	Random sampling	No	1048	Women > 18 years old	–	18 to 90	Community (rural)	Questionnaire, pelvic examination performed	UDI-6	ICS	42.1	21.3	40.6	38.1
Mikou et al. [27]	Morocco	Cross-sectional study	Random sampling	No	1000	Women > 18 years old	–	18 to 85	Community	Questionnaire	No	–	27.1	42.8	49.4	7.8
Mourad et al. [22]	Egypt	Cross-sectional study	Random sampling	No	1853	Women < 18 years old	–	≥ 18	Community	Questionnaire	No	ICS	27	22.2	22.2	44.4
Niang et al. [35]	Mauritania, Senegal, Chad	Cross-sectional study	Nonrandom sampling	No	2070	Women > 18 years old	28	Adulte	Community	Questionnaire	No	–	17.7	28.6	38.4	33
Ofori et al. [34]	Ghana	Cross-sectional study	Nonrandom sampling	Yes	400	Women who visited the clinic	42.7 ± 12.5	19 to 88	Hospital	Questionnaire	ICIQ-UI SF	–	12	33.3	22.9	20.8
Ojengbede et al. [18]	Nigeria	Cross-sectional study	Random sampling	No	5001	Women > 18 years old	33.2 ± 14.7	≥ 18	Community (rural and urban)	Questionnaire	No	–	2.8 (2.6–3.)	–	–	–
Terefe et al. [24]	Ethiopia	Cross-sectional study	Random sampling	Yes	275	Women who visited the clinic > 18 years old	33.3 ± 10.8	–	Hospital	Questionnaire	No	–	9.3 (5.6–13)	–	–	

*ICIQ-UI SF* International Consultation on Incontinence Questionnaire–Urinary Incontinence Short Form, *QUID* Questionnaire for Urinary Incontinence Diagnosis, *PFDI-20* Pelvic Floor Disability Index, *USP* urinary symptoms profile, *ICS* International Continence Society, *UI* urinary incontinence, *UUI* urgency urinary incontinence, *SUI* stress urinary incontinence, *MUI* mixed urinary incontinence

This systematic review and meta-analysis bring together studies conducted in different African countries. It covers studies on the prevalence of UI among females aged at least 18 years (mean ranged from 28 to 70.7 years). Studies on UI in Africa have been conducted in various countries: 6 in Nigeria (27.3%) [14–19], 3 (13.6%) in Egypt [20–22] and Ethiopia [23–25], 2 (9.1%) in Morocco [26, 27], and 1 (4.5%) in other countries: Tunisia [28], Rwanda [29], Liberia [30], Burkina Faso [31], South Africa [32], Tanzania [33], and Ghana [34]. One study was conducted in three countries simultaneously (Mauritania, Senegal, and Chad) [35].

All studies were cross-sectional, with 13 (59.1%) studies using random sampling and 9 using a nonrandomized method. They were conducted in the community (15 studies: 2 in rural areas, 5 in urban areas, 3 in both urban and rural areas, and 5 in unspecified areas) and in hospitals.

In the hospital setting, the studies were conducted either with women who visited the gynecology clinic and/or their accompanying individuals (five studies), or with health care professionals from the hospital [28], or among frail elderly women older than 60 years who were hospitalized [20].

Eight studies presented the sample size, whereas 14 did not. The sample size varied from 99 to 5001 women.

To determine the prevalence of female UI, less than half of the studies used validated questionnaires, in particular, the International Consultation on Incontinence Questionnaire-Urinary Incontinence Short Form (ICIQ-UI SF) in 4 studies [14, 20, 29, 34], the Pelvic Floor Disability Index (PFDI-20) [30] or the Urinary Distress Inventory-short form questionnaire (UDI-6) in 3 studies [21, 33], the Questionnaire for Urinary Incontinence Diagnosis (QUID) [16], and the Urinary Symptoms Profile (USP) in 1 study each [28], whereas 13 studies used nonvalidated questionnaires.

Few studies (22.7%) have used the definition of UI provided by the International Continence Society (ICS) [21–23, 32, 33], which defines it as “any complaint of involuntary urine loss” [1]. The majority (77.3%) of studies used other types of definitions.

### Prevalence of Urinary Incontinence

**Fig. 3:**
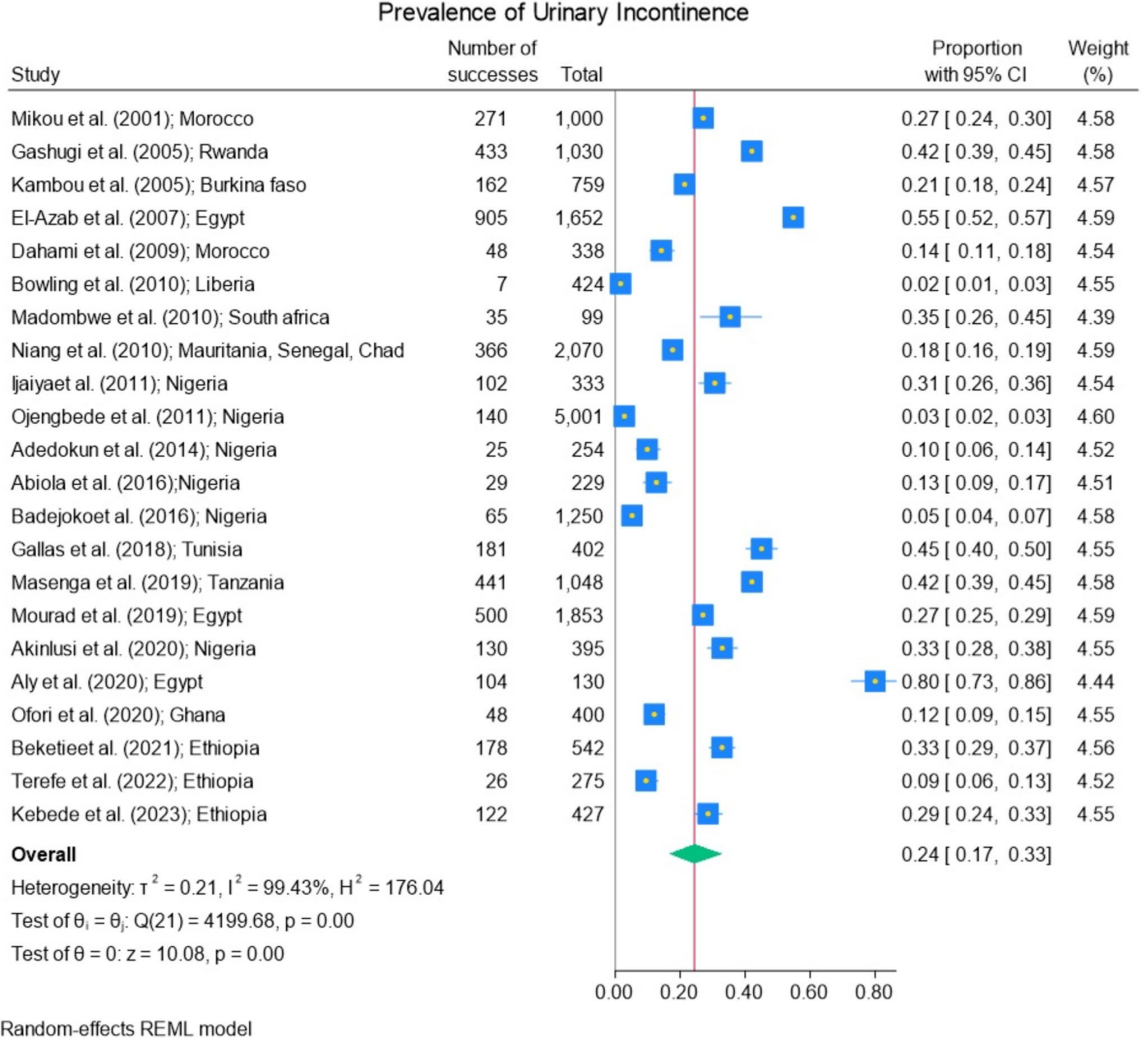
Prevalence of urinary incontinence

**Fig. 4:**
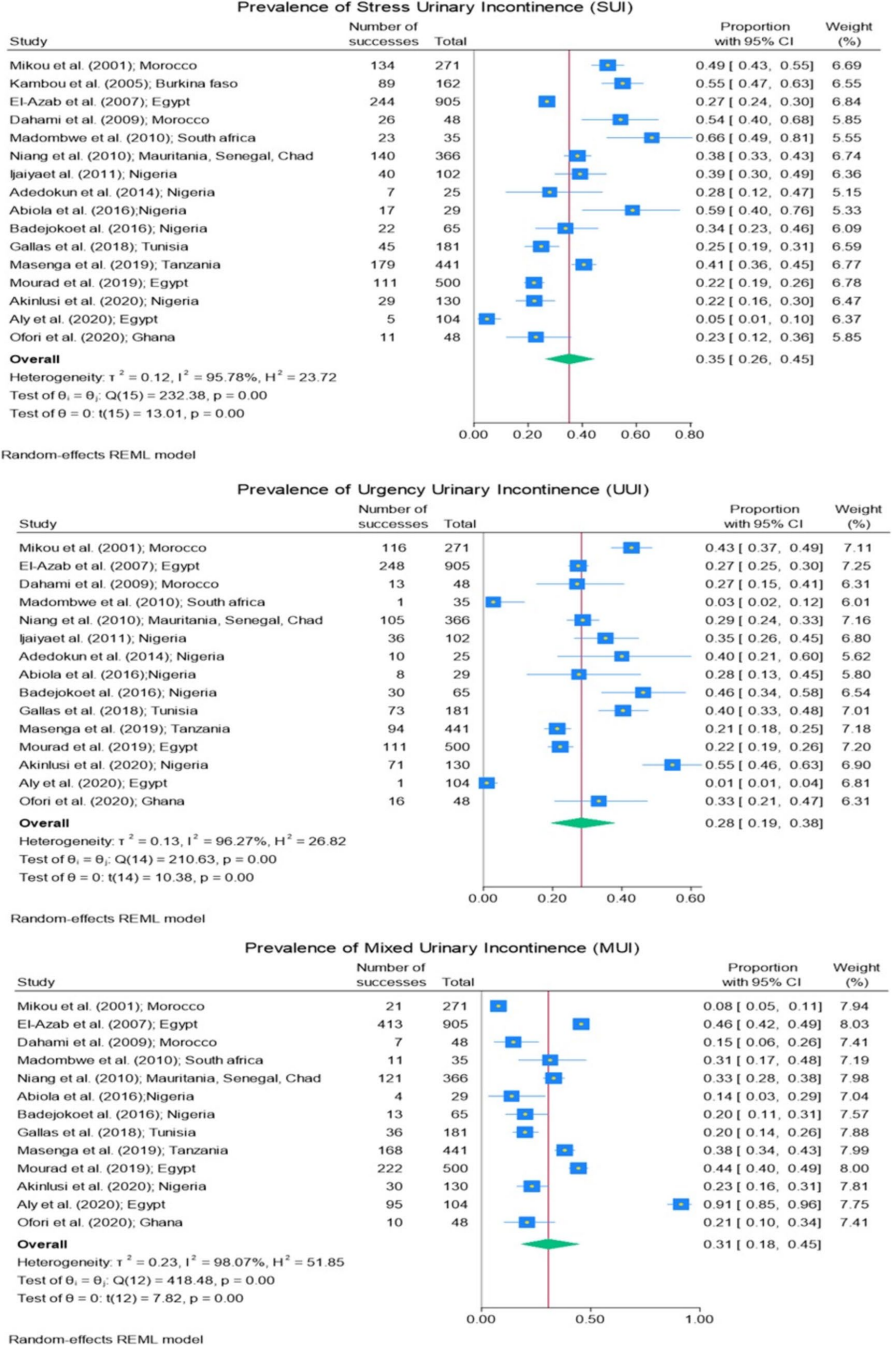
Prevalence of types of urinary incontinence

Figures 3 and 4 illustrate the pooled prevalence of UI and its types as well as that of each study. The pooled prevalence of UI was 24% (95% CI 17–33%) ranging from 2 to 80%. The pooled prevalence of the different types of UI, including UUI, SUI, and MUI, were 28% (95% CI 19–38%; ranging from 1 to 55%), 35% (95% CI 26–45%; ranging from 5 to 66%), and 31% (95% CI 18–45%; ranging from 8 to 91%) respectively.

### Prevalence of Urinary Incontinence According to Geographical Region

**Fig. 5:**
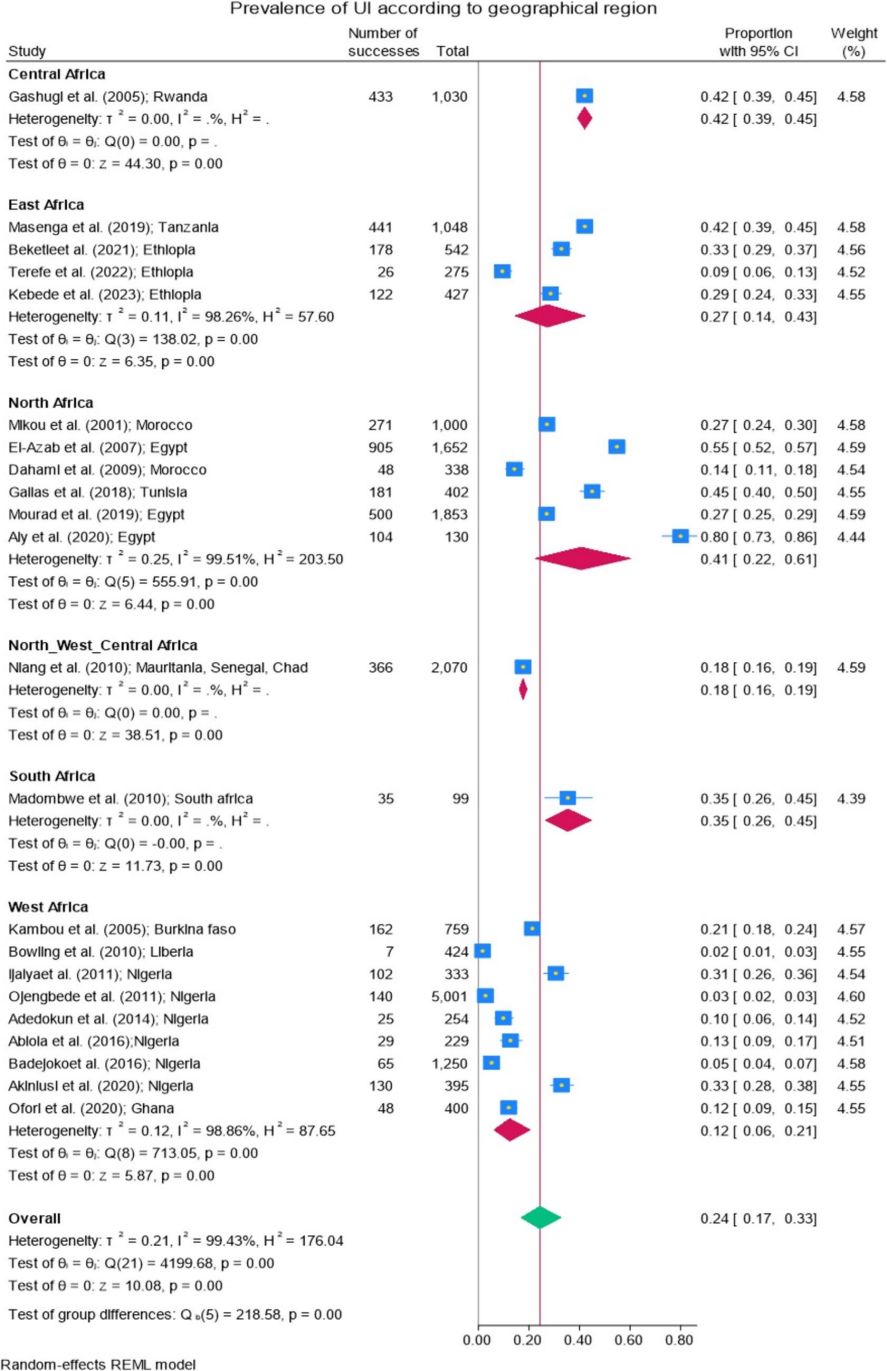

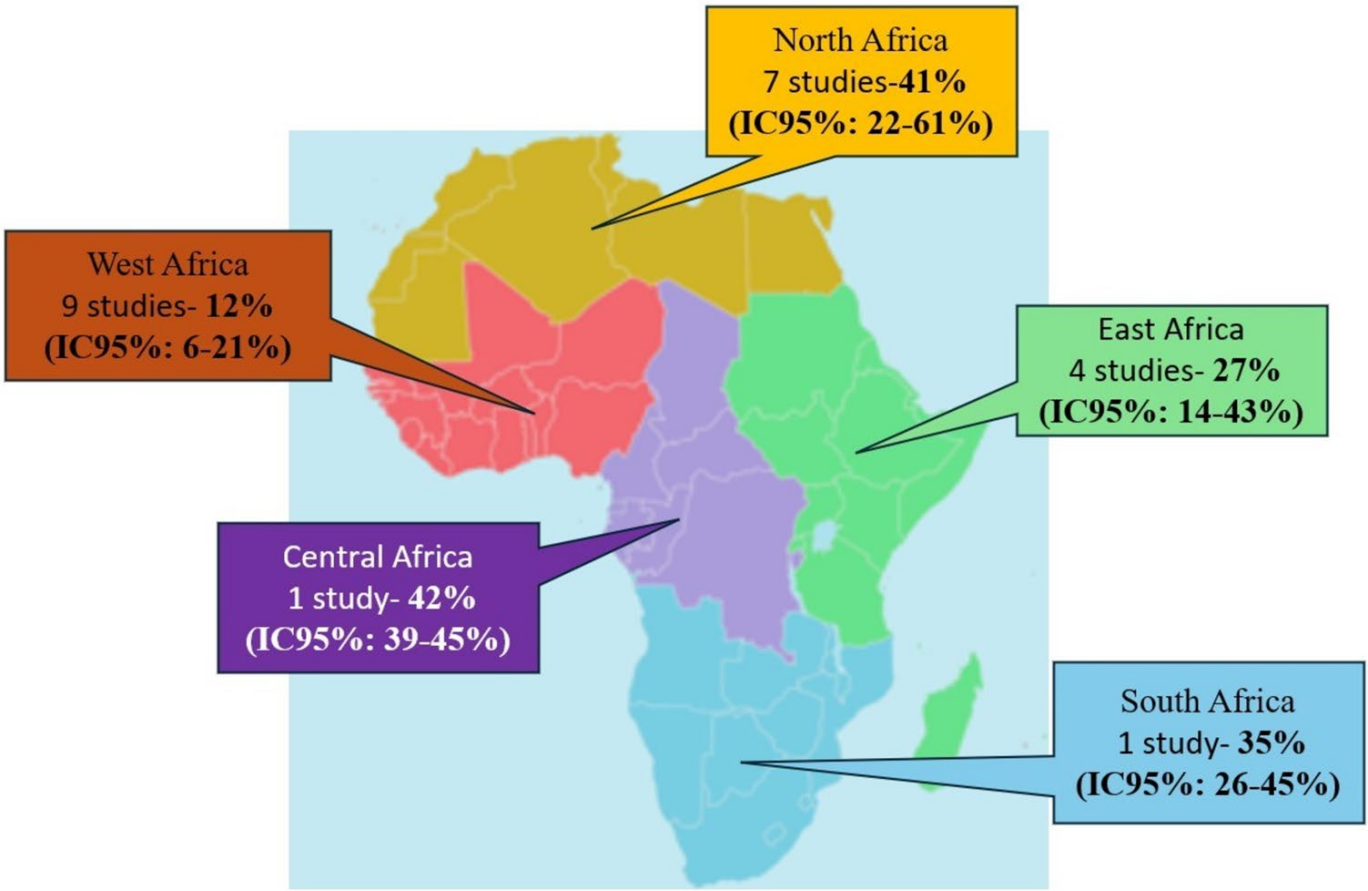
**a** Prevalence of urinary incontinence according to African regions. **b** Map of estimated prevalence of female urinary incontinence across African regions

The pooled prevalence of UI based on geographical region was studied in five regions of Africa: Central Africa, East Africa, West Africa, North Africa, and South Africa (Fig. 5). A difference between the regions was observed (*p* < 0.001), with a higher prevalence in Central Africa and North Africa (Fig. 5a). A statistical difference of prevalence between the regions was observed (*p* < 0.001), with a higher prevalence in Central Africa and North Africa (Fig. 5a).

### Prevalence According to Racial Distribution (White Africa and Black Africa)

**Fig. 6:**
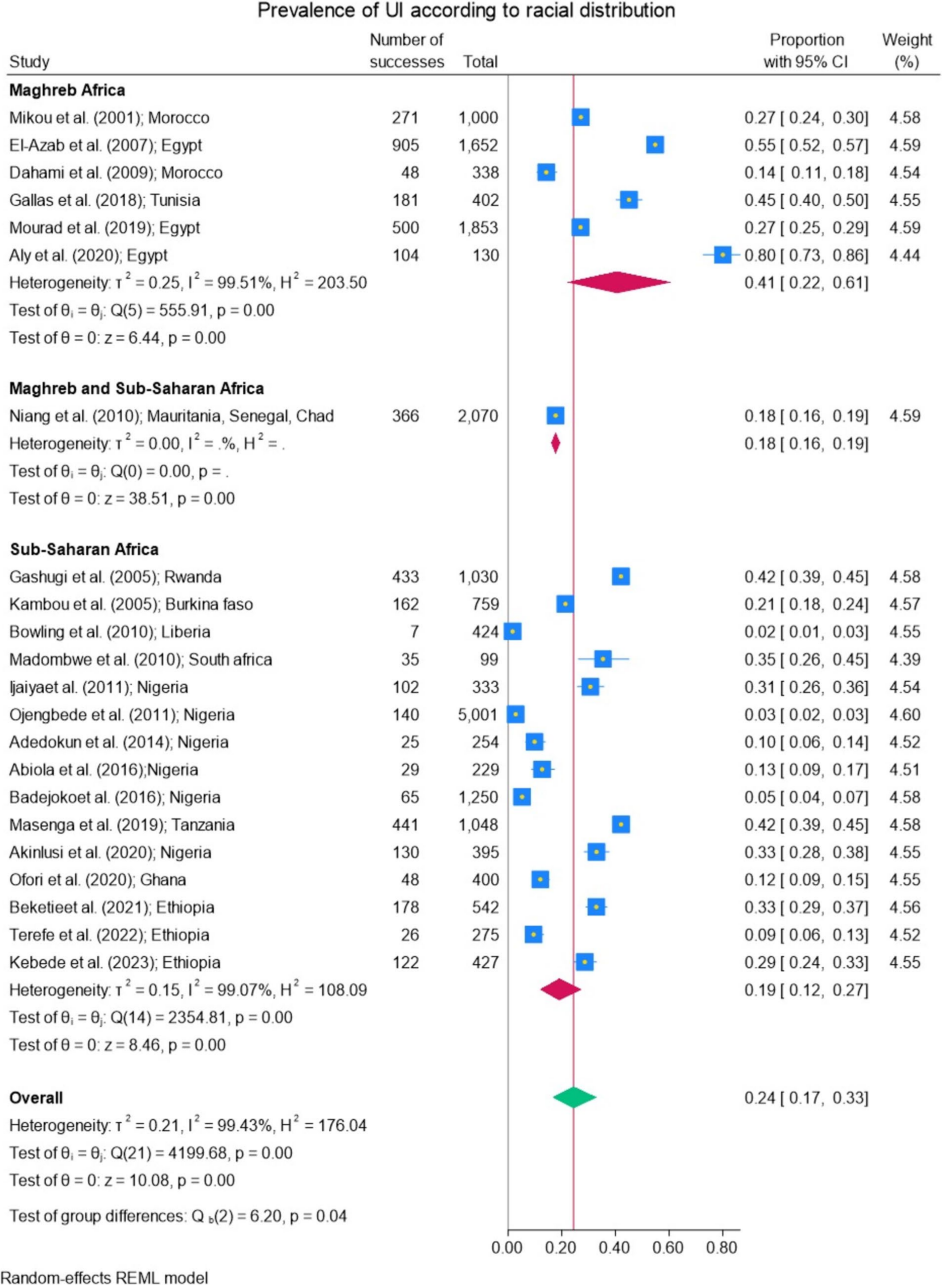
**a** Prevalence of urinary incontinence (UI) according to racial distribution. **b** Prevalence of stress urinary incontinence (SUI) according to racial distribution. **c** Prevalence of urgency urinary incontinence (UUI) according to racial distribution. **d** Prevalence of mixed urinary incontinence (MUI) according to racial distribution

The prevalence of UI according to racial distribution was also investigated (Fig. 6, Table 4). It was 41% (95% CI 22−61%) for white Africa or the Maghreb African region (including 6 studies) and 19% (95% CI 12−27%) for Black Africa or sub-Saharan Africa region (15 studies included).

**Table 4:** Summary of subgroup analyses for urinary incontinence (UI) in Africa

Variables (studies number)	Prevalence, % (95% CI)	*p* value of Z test (groups)	*p* value of Q test for group (heterogeneity)	I^2^ (inconsistency) for group	*p* value for test of group differences
Prevalence of UI based on the use of validated questionnaires					
No (13)	21 (15–28)	< 0.001	< 0.001	98.6	0.39
Yes (9)	29 (13–50)	< 0.001	< 0.001	99.6	
Prevalence of UI based on method of data collection					
Questionnaire (20)	21 (15–28)	< 0.001	< 0.001	99.2	0.04
Questionnaire and pelvic examination performed (2)	62 (24–93)	< 0.001	< 0.001	98.6	
Prevalence of UI based on study on UI isolated or associated with other PFD					
With other PFD (6)	18 (7–33)	< 0.001	< 0.001	98.9	0.30
UI isolated (16)	27 (18–38)	< 0	< 0.001	99.5	
Prevalence of UI based on urban or rural environment					
Both (3)	25 (2–61)	< 0.001	< 0.001	99.8	
Rural(2)	26 (4–58)	< 0.001	< 0.001	98.8	< 0.001
Urban (5)	23 (12–36)	< 0.001	< 0.001	97.8	
Semi-urban (1)	5 (4–7)	< 0.001			
Prevalence of UI over decade					
2000–2010 (8)	25 (13–38)	< 0.001	< 0.001	99.3	0.98
2011–2020 (11)	25 (12–39)	< 0.001	< 0001	99.5	
2021–2023 (3)	23 (10–39)	< 0.001	< 0.001	97.5	
Prevalence based on study site					
Hospital (1)	80 (73–86)	< 0.001			
Specific community in the hospital (8)	19 (11–30)	< 0.001	< 0001	98.3	< 0.001
Community (13)	24 (15–34)	< 0.001	< 0.001	99.4	
Prevalence of SUI according to racial distribution					
Maghreb Africa (5)	28 (15–45)	< 0.001	< 0.001	97.9	0.46
Sub-Saharan Africa (9)	40 (30–50)	< 0.001	< 0.001	88.7	
Both (1)	38 (33–43)	< 0.001			
Prevalence of UUI according to racial distribution					
Maghreb Africa (6)	25 (11–41)	< 0.001	< 0.001	98	0.77
Sub-Saharan Africa (8)	31 (20–44)	< 0.001	< 0.001	91.7	
Both (1)	29 (24–33)	< 0.001			
Prevalence of MUI according to racial distribution					
Maghreb Africa (6)	36 (13–64)	< 0.001	< 0.001	99.3	0.26
Sub-Saharan Africa (6)	25 (18–33)	< 0.001	< 0.001	72.6	
Both (1)	33 (28–38)	< 0.001			
Prevalence of UI based on the sampling method					
Nonrandom sampling (9)	21 (9–38)	< 0.001	< 0.001	99.4	0.55
Random sampling (13)	27 (18–37)	< 0.001	< 0.001	99.3	
Prevalence based on calculating the sample size					
No (14)	27 (16–39)	< 0.001	< 0.001	99.6	0.43
Yes (8)	20 (12–31)	< 0.001	< 0.001	98.2	

*PFD* pelvic floor dysfunction, *SUI* stress urinary incontinence, *UUI* urgency urinary incontinence, *MUI* mixed urinary incontinence

A difference between racial distribution was observed (*p* < 0.04) with major prevalence of UI for the Maghreb African region (Fig. 6a). No significant difference was found for SUI (Fig. 6b), UUI (Fig. 6c), and MUI (Fig. 6d) according to racial distribution among the studies that addressed the types of urinary incontinence.

### Prevalence of Urinary Incontinence Based on the International Continence Society Definition of Incontinence

**Fig. 7:**
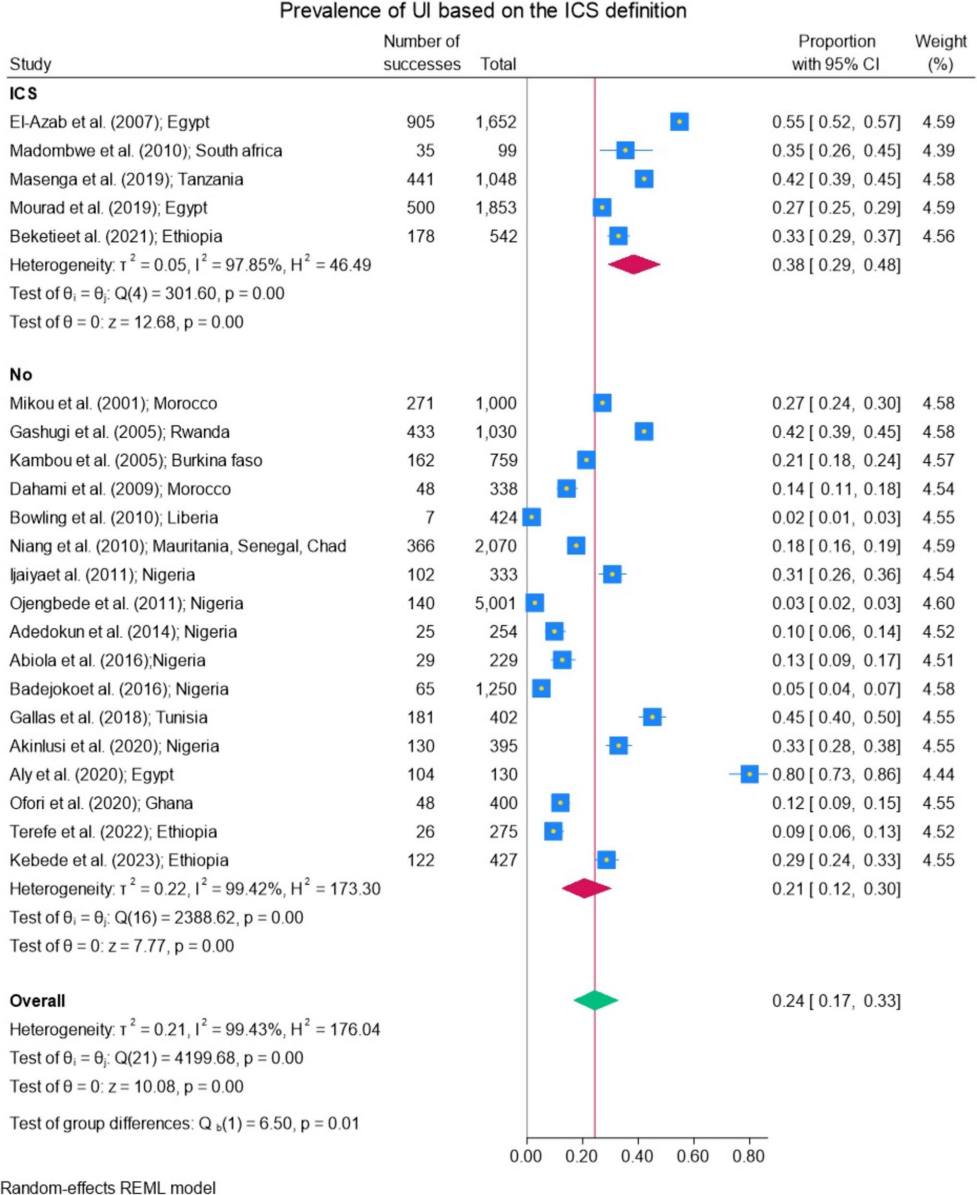
Prevalence of urinary incontinence (UI) based on the International Continence Society (ICS) definition

A higher prevalence of urinary UI was observed at 38% (95% CI 29−48%) when the ICS definition was used for diagnosis. In contrast, when a different definition was applied, the prevalence was lower, at 21% (95% CI 12−30%) (*p* = 0.01; Fig. 7). Some authors defined UI either as “involuntary loss of urine that has become a social or hygienic problem” [17] or by asking the question “Have you experienced any involuntary loss of urine in the past month?” [34]. For others, the definition of UI was not specified.

Table 4 summarizes the results of the meta-analysis on the prevalence of UI, stratified by various influencing factors, including the use of validated versus nonvalidated questionnaires, sampling method, sample-size calculation, rural versus nonrural settings, and study site (hospital-based versus community-based), etc. (Table 4).

### Meta-Analysis

Height graphs (Figs. 3, 4, 5a, 6, and 7) were created to illustrate the results of the meta-analysis. Cochran Q test was used to assess the heterogeneity of the studies included in the meta-analysis for UI, types of UI, and their characteristics of different subgroups. The I^2^ test was used for assessing inconsistency between these studies. The *p* values for the Cochran Q test for all categories of UI used realized owing to the random-effects model were < 0.001 and their I^2^ test ranged from 72.6 to 99.8%. It means that the studies’ prevalence of UI from different subgroup characteristics demonstrated high heterogeneity.

### Risk of Bias Across Studies

**Fig. 8:**
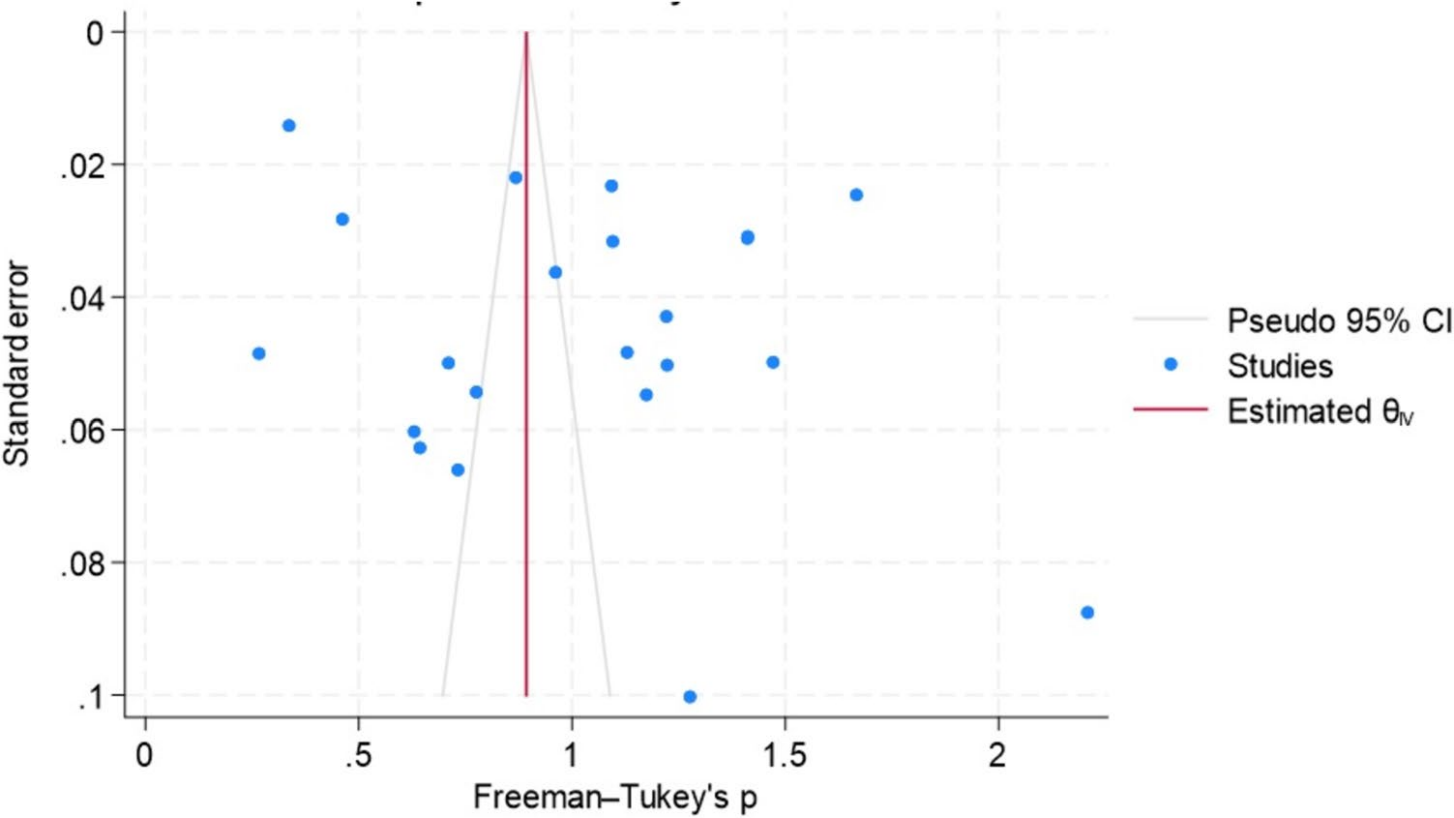
Funnel plot of urinary incontinence

By funnel-plot analysis (Fig. 8), the distribution scheme was asymmetric around the central axis and scattered, with many studies falling outside the 95% CI zone. This explains the high heterogeneity and bias among the studies analyzed.

## Discussion

### Prevalence of Urinary Incontinence

This meta-analysis shows that the pooled UI prevalence was high, with a rate of 24% (95% CI 17—33%) ranging from 2 to 80%. However, there is considerable heterogeneity between different studies compiled in this systematic review depending on several parameters such as UI definition, methodology, and population studied.

This prevalence corroborates that found in the systematic review/meta-analysis by Mostafaei et al. [3] and in the review of prevalence by Walker and Gunasekera [36], all carried out in the developing world. Their respective values were 25.7% (95% CI 22.3–29.5%), with extremes ranging from 2.8 to 57.7% and 28.7%, with extremes ranging from 5.2 to 70.8%. It is also close to the prevalence found in the systematic review by Islam et al. [37] of community-dwelling women in low- and middle-income countries, which was 30% (95% CI 25–35%).

In terms of geographical regions, UI was most prevalent in Central Africa, with a rate of 42% (95% CI 39–45%), based on data from a single country, Rwanda [29]. In North Africa, the prevalence was 41% (95% CI 22–61%), with the highest rate recorded at 80% (95% CI 73–86%) in the study by Aly et al. conducted in Egypt [20]. UI was less common in West Africa, where the prevalence was 12% (95% CI 6–21%).

Racial disparities in the prevalence of female UI were observed across regions. In Maghreb Africa, the prevalence was notably higher, at 41% (95% CI 22–61%), with rates varying from 14% [26] to 80% [20]. In contrast, sub-Saharan Africa had a lower prevalence of 19% (95% CI 12–27%), with rates ranging from 2% [30] to 42% [29]. Similar findings were reported in the study by Mostafaei et al., whereas studies conducted in the Middle East and North Africa region showed a higher pooled prevalence (37.3%, 95% CI 25.8–50.5%) compared with those in sub-Saharan Africa (4.6%, 95% CI 1.7–12.3%) [3].

Mostafaei et al. [3] identify several factors that may explain regional variations in the prevalence of UI, including cultural and racial differences. Other contributing factors include the social impact of UI, feelings of shame, and a lack of confidence in the health care system. Additionally, the lack of knowledge and understanding of UI as a health issue may discourage women from seeking help. Some women may choose to conceal their condition, whereas others may perceive it as a natural part of aging [38]. However, the reasons for not seeking care are multifactorial.

When the ICS definition was used to diagnose UI, the prevalence was higher, at 38% (95% CI 29–48%), compared with when another definition was applied, which resulted in a prevalence of 21% (95% CI 12–30%). This heterogeneity in prevalence data can be attributed to the use of different definitions for diagnosing UI, which may capture varying aspects of the condition. The variation in diagnostic criteria likely leads to discrepancies in the reported prevalence, as certain definitions may encompass a broader range of symptoms or severity levels. The ICS definition may result in higher prevalence rates in epidemiological studies because it is easier to apply in clinical practice and research as it is broader and more inclusive, focusing on the symptom (urinary leakage) rather than the underlying cause. This approach allows for a more standardized and practical identification of UI, making it applicable to a wider range of patients and research settings.

Despite the significant heterogeneity observed, this systematic review and meta-analysis generally showed that the pooled prevalence appeared higher when the study was conducted in North Africa (Maghreb) or Central Africa, and when the ICS definition of UI was used. However, the analyses found that regardless of the sampling method used, whether the sample size was calculated or not, or whether validated questionnaires were employed, the prevalence was statistically similar. Nevertheless, the significant heterogeneity observed suggests that there might be variability in the methodological strategies employed across studies for estimating the prevalence of UI. This variation could stem from differences in study design, population characteristics, and diagnostic criteria, all of which may influence the reported prevalence.

Regarding the pooled prevalence of UI types, SUI was the most frequent type compared with UUI, with a prevalence of 35% (95% CI 26–45%) and 28% (95% CI 19–38%) respectively.

The same observation was made in the study by Mostafaei et al., where they examined the prevalence of different types of UI in developing countries, reporting SUI and UUI prevalence of 12.6% and 5.3% respectively [3]. However, this trend varied across studies conducted in Africa, as 9 studies showed a predominance of SUI, whereas 6 other studies [15, 16, 19, 21, 28, 34] reported a predominance of UUI. In addition, no significant difference in the prevalence of UI types was found when comparing studies conducted in white Africa and those conducted in Black Africa. This suggests that, despite potential cultural and regional differences, the distribution of UI types may be similar across these regions.

The systematic review conducted by Gonzalez et al. [39] on racial/ethnic disparities points out that most population-based studies suggest that racial differences might play a predictive role in UI. In general, most studies showed a significant predominance of SUI over UUI in white women compared with African American women [39–45]. Thom et al. [46], in their population-based study conducted in the USA, pointed out that fewer Black women had SUI than all the other groups studied (Asian, white, and Hispanic women), but had the highest prevalence of UUI.

It is therefore difficult to draw conclusions on the racial predominance of one type of UI, given the heterogeneity observed across studies. This variation may reflect differences in study populations, diagnostic criteria, and regional factors, which could influence the prevalence and distribution of UI types across racial groups. Further research with standardized methodologies and larger sample sizes may help to clarify these patterns.

Thus, the studies on the prevalence of UI and its types, considering the different subgroups included in this systematic review and meta-analysis, have demonstrated considerable heterogeneity. Similarly, the asymmetry observed in the funnel-plot analysis suggests the probable existence of publication or methodological bias.

This indicates that not all studies used the same diagnostic tools for UI, nor did they follow the same methodological approach (sample size and type, questionnaires, population, ethnic groups, etc.). These factors complicate the interpretation of the data from this meta-analysis, and conclusions should therefore be drawn with caution.

### Strengths and Limitations

To our knowledge, this systematic review and meta-analysis are the first on the prevalence of UI in adult women on the entire African continent, covering a long period from 2000 to 2023.

The databases used may constitute a selection bias, as they group together more articles mainly published in English than in French, and some African articles are not published in them; this is why we resorted to The African JOL, where some published African articles are stored. The use of different definitions of UI and methodologies in conducting these studies constituted a bias, resulting in significant heterogeneity in the analysis of the prevalence of UI among women in Africa. Additionally, the methodological quality of the studies was not consistent, with more than half of the studies being of low quality.

### Recommendations for Future Research

In view of this considerable heterogeneity in the analysis of UI prevalence rates in Africa, future studies should use the same definition recognized by international scientists, the same validated questionnaires, the same methodological approach, in the same population, and in the same age group. It would be better to sensitize investigators and the community to the symptomatology of UI and its types, and if possible, the translation of the validated questionnaire into the language spoken and mastered by the community studied. For further research, it would also be valuable to focus on the associated factors, behaviors, and trends in health care seeking, in addition to prevalence.

## Conclusion

Urinary incontinence affects one-quarter of adult women in Africa. This prevalence appears to be higher in North Africa (Maghreb) and Central Africa when studies used the ICS definition of UI. The prevalence remains consistent regardless of the questionnaire used, whether validated or not, or the sampling method employed. However, the high degree of heterogeneity observed in the meta-analysis prevents confirmation of these trends based on various characteristics, owing to differences in methodological approaches and the definitions of UI used in the selected studies.

## Data Availability

All data analyzed in this study were extracted from previously published articles. No new data were generated.
